# Anatomy at the threshold: Teaching the human body in a hybrid age

**DOI:** 10.1002/ase.70121

**Published:** 2025-09-07

**Authors:** Katia Cortese, Paola Falletta

**Affiliations:** ^1^ DIMES, Department of Experimental Medicine, Human Anatomy University of Genoa Genoa Italy; ^2^ Human Anatomy Vita‐Salute San Raffaele University Milan Italy; ^3^ Experimental Imaging Center, IRCCS Ospedale San Raffaele Milan Italy

**Keywords:** anatomy education, artificial intelligence, critical medical humanities, human enhancement, hybrid body

## Abstract

As emerging technologies reshape both the body and how we represent it, anatomical education stands at a threshold. Virtual dissection tools, AI‐generated images, and immersive platforms are redefining how students learn anatomy, while real‐world bodies are becoming hybridized through implants, neural interfaces, and bioengineered components. This Viewpoint explores what it means to teach human anatomy when the body is no longer entirely natural, and the image is no longer entirely real. Based on recent evidence and educational reflections, it suggests that anatomy can serve as a critical human science, one that goes beyond structural knowledge, encouraging students to develop visual literacy, structural reasoning, and ethical awareness. As experiences with donated bodies are replaced with digital models, students risk losing contact with the lived, variable, and vulnerable aspects of the human form. Yet, rather than resisting change, anatomists can respond by integrating new tools within a pedagogical model grounded in presence and meaning. In an age where biology and technology are converging with unexpected speed, anatomy offers a powerful lens to question not only how bodies work, but what bodies mean. The role of the anatomist is therefore both conservative and visionary: to hold the line of deep biological knowledge, while opening the door to critical engagement with the hybrid human condition.

## INTRODUCTION

**FIGURE 1:**
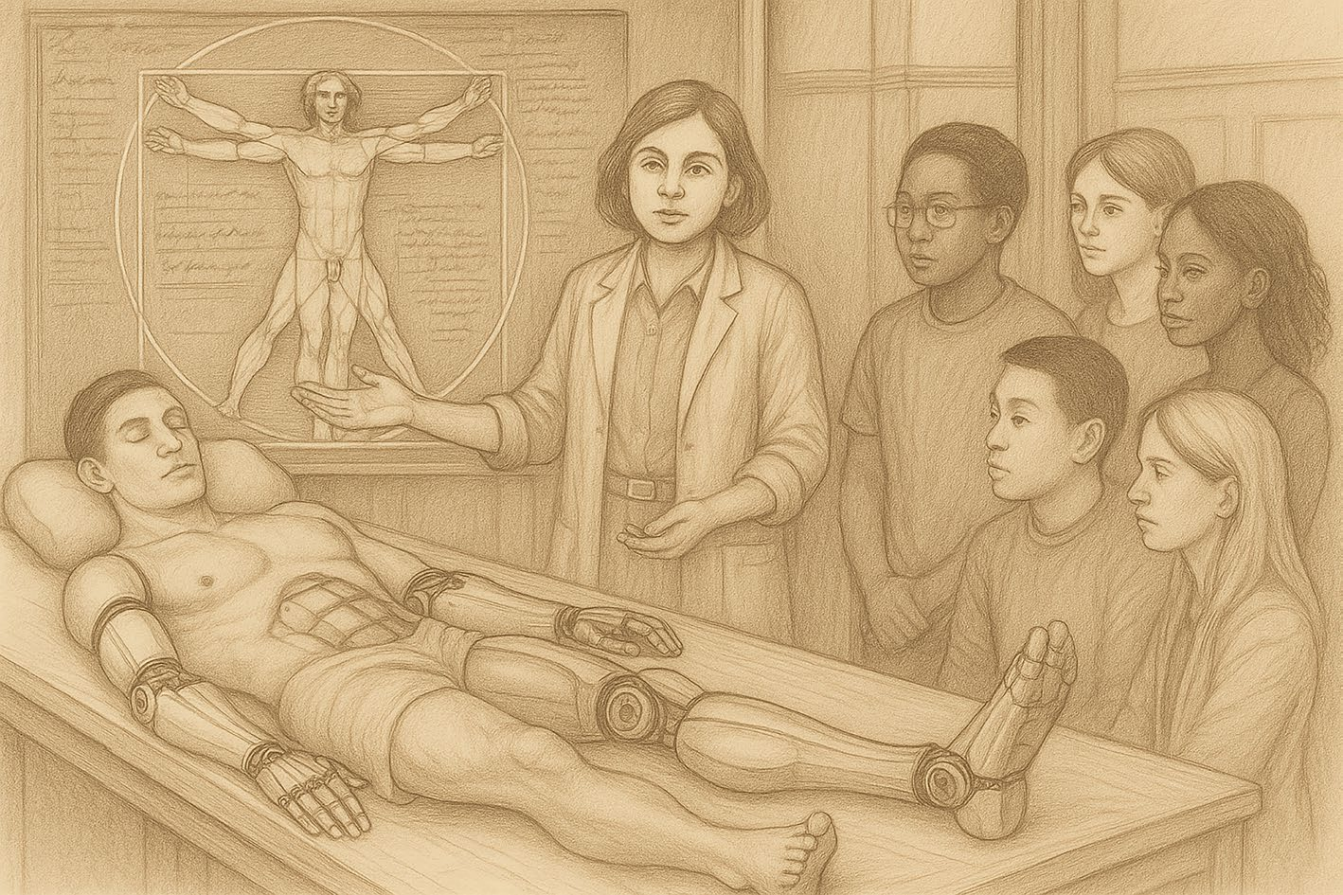
A pencil‐style illustration depicting a modern anatomy lesson where classical human anatomy meets contemporary technological frontiers. The partially robotic body on the dissection table symbolizes the convergence of biological and engineered forms, inviting reflection on the enduring value of hands‐on anatomical study even as education evolves alongside technological innovation. The Vitruvian Man on the blackboard anchors the scene in historical ideals, underscoring how traditional frameworks continue to inform interpretations of an increasingly hybrid human form. *Compliance statement*: This figure was created using an AI‐assisted image generation tool (DALL·E), guided by iterative text prompts to achieve anatomical accuracy, integrate hybrid human‐machine elements, and replicate a graphite drawing style. No human or patient images were used.

What does it mean to teach human anatomy when the very idea of the human body is being redefined, not only by biology, but also by technology? (Figure 1). As anatomists, we have spent many years guiding students through the architecture of the body, we have worked with real tissues, glass slides, and electron micrographs, convinced that form is not just structure, but carries meaning, a view we recently elaborated in the context of scientific interpretation.^1^ Today, however, we are facing a threshold moment in anatomy education, where the tension between synthetic simulation and embodied experience is growing sharper. Curricular goals are shifting beyond structural knowledge to include competencies such as interpreting probabilistic atlases and reasoning through digital twins, reflecting how anatomy is increasingly mediated by data and algorithms. This is not only an educational transition, as we integrate virtual dissection tables, AI‐driven platforms, and immersive 3D tools, but also an anthropological one.^2^ Anatomists have long adapted to evolving bodies, from implants to organ transplants, but today the hybrid body ‐ at once biological, technological, and data‐defined ‐ emerges as a central object of both anatomy education and healthcare. This cultural acceleration and symbolic weight of today's hybridization and automation require a renewed pedagogical perspective. Artificial intelligence is changing how we generate, visualize, and interpret knowledge. Algorithms now map and label anatomical structures, while AI‐generated images are entering not only in education, but also in scientific publications. This shift has raised concerns about the reliability of synthetic images, as discussed by Bucci and Parini,^3^ who warn of a “synthetic image crisis” in biomedical research: a situation where entirely fabricated but plausible images, produced without experimental data, may compromise trust, reproducibility, and the integrity of image‐based evidence. The authors call for urgent updates to editorial and peer‐review protocols, including stricter validation processes and transparency measures, to ensure that digital images remain accurate and trustworthy representations. Beyond the classroom, AI‐based diagnostics and robotic surgery are reshaping how we interact with the human body.^4^, ^5^, ^6^ These shifts not only affect how we teach but they challenge what we are teaching and the epistemological assumptions behind it. At the same time, the biological body itself is changing. As implants, sensors, and engineered components become part of clinical reality, the object we study in anatomy is becoming less stable, more personalized, and more hybrid.^7^ This Viewpoint arises from this double transformation. It is a ponderation on what it means to teach anatomy when the body is no longer entirely natural, and the image is no longer entirely real. As the lines between human and machine, between physical and virtual, become less defined, anatomy education should promote a critical understanding that helps students navigate between synthetic representations and the real, lived body.

## THE SIGNIFICANCE OF ANATOMY TEACHING IN THE ERA OF THE HYBRID BODY

The anatomist of today acquires the novel role of preparing students for a landscape in which the “hybrid body” ‐ a human form simultaneously biological, technological, and data‐driven ‐ has become the default clinical reality. The broad concept of the hybrid body we advance here encompasses several overlapping meanings, which can be situated within existing academic frameworks and usefully articulated as a tripartite construct. Ontological hybridity denotes direct biological‐technological integrations such as cochlear implants, brain‐computer interfaces, and bioengineered organs, situating the patient “as an ontological go‐between” that blurs conventional boundaries between natural and artificial structures.^8^ Representational hybridity captures how anatomical understanding increasingly relies on digital surrogates. For instance, 3D printing now serves as a powerful pedagogical tool for teaching human anatomy, enhancing tactile and spatial learning.^9^ Moreover, the rapidly evolving field of synthetic data generation in medical imaging, such as metadata‐conditioned models spanning MRI, CT and plain film radiographs, raises new questions about technical and ethical challenges, such as ensuring realism and diversity, and preserving patient identity.^10^ At the frontier, anatomical digital twins offer perpetual, multimodal reconstructions of patient‐specific structures that support both medical education^11^ and personalized surgical planning.^12^ The growing reliance on representational hybridity raises important questions about authenticity, reliability, and the potential erosion of anatomical meaning. Indeed, as Bucci and Parini^3^ caution in describing the emerging “synthetic image crisis,” this turn toward algorithmically generated anatomy also carries profound risks: it may compromise the epistemic trust historically vested in anatomical images and challenge the ability of students to distinguish empirical evidence from purely computational constructs. In fact, Lazarus et al.^13^ elucidate how the shift toward computationally generated anatomy demands rigorous reflection on the perils of AI in anatomical education. Moreover, Cornwall et al.^14^ frame a cautionary discussion on how AI‐generated images might disrupt the epistemic integrity of anatomical illustration. These developments have been systematically mapped by the National Academies,^15^ which identify foundational research gaps and call for frameworks to ensure that virtual representations remain scientifically robust and ethically accountable.

Finally, epistemic hybridity captures a profound transformation in how anatomical knowledge itself is constructed: moving away from deterministic models toward probabilistic, multi‐scale, data‐centric frameworks. Here, “structure” emerges as a statistical reconstruction from vast clouds of longitudinal and multimodal data, spanning from histology to advanced automated MRI segmentation,^16^ highlighting a core aspect of epistemic hybridity where anatomical “reality” is established through aggregated and computationally harmonized datasets.^17^ Landmark initiatives such as HuBMAP have created high‐resolution, multi‐organ reference atlases that integrate diverse data types to map human variability with unprecedented depth.^18^ These developments are accompanied by rigorous methodologies for validation, uncertainty quantification, and explainability to ensure that digital twins and predictive anatomical models are both scientifically robust and clinically meaningful.^19^, ^20^ As Leonelli^21^ argues, this data‐centric turn represents not merely a technical evolution but a philosophical shift, compelling educators, and practitioners to reconsider what counts as anatomical “truth” in an era when structure is increasingly inferred rather than directly observed.

Recognizing these three constructs clarifies why traditional dissection remains pedagogically irreplaceable, anchoring students in the material reality of embodied variation, yet also why curricula must now integrate digital‐twin reasoning, AI‐mediated image provenance, and critical reflection on human–technology entanglement. In doing so, anatomy teaching retains its historic role as the foundational human science while preparing learners to navigate and ethically interrogate the fully hybrid human condition that awaits them at the bedside and in the operating theater.

## THE CHANGING ANATOMY CLASSROOM

Over the past two decades, and even more after the COVID‐19 pandemic, anatomical education has undergone a profound transformation, driven by both pedagogical innovation and digital technology.^22^, ^23^, ^24^, ^25^ Traditional dissection of deceased donors,^26^ long considered the gold standard, is now increasingly complemented, or in some cases replaced, by immersive and interactive tools. These include 3D visualization apps, virtualKassanos dissection tables like the Anatomage Table, and technologies based on augmented (AR) and virtual reality (VR).^27^ A growing body of evidence supports the educational value of these innovations. For example, an analysis of 27 studies; n ≈ 2000, found that extended reality (XR) significantly improved anatomical knowledge, particularly when used to supplement rather than replace traditional methods (pooled SMD ≈ 0.52).^28^ Student responses are generally enthusiastic. Another study reported that learners using VR platforms such as 3D Organon felt they had a better grasp of anatomy, especially when these technologies were integrated with lectures and dissection.^29^ Similarly, a recent study documented high satisfaction across undergraduate courses, while also noting concerns about long‐term outcomes and practical issues like cost.^30^ However, this enthusiasm must not obscure important pedagogical questions. Digital anatomy is not just a translation of older methods into a new format; it is an emerging field that requires its own pedagogical framework. These tools offer flexibility and interactivity, but they cannot replicate the tactile, emotional, and sensorial experience of working with a real body.^31^ Indeed, training using deceased human bodies significantly enhances surgical confidence, competence, and anatomical understanding across specialties, from trauma surgery (C‐BEST program) to thoracic surgery (CALNA program) and abdominal wall reconstruction^32^, ^33^ providing irreplaceable experiential learning that surpasses virtual models in realism, spatial comprehension, and psychomotor skill acquisition. A critical and balanced approach is therefore essential, guided by explicit comparative criteria, such as competence threshold, cost considerations, and access equity, to determine when digital advantages outweigh embodied losses. Digital platforms enhance spatial understanding and access, but they may also flatten the complexity of the human body into idealized, manipulable models. This concern is at the heart of recent ethical discussions on whether AI‐generated human simulations should replace donor‐based dissection, highlighting tensions between efficiency, realism, and educational value.^34^ The shift from “scalpel to screen” can speed up learning, but it risks weakening the deep, embodied engagement that supports clinical empathy and professional development.^35^ Rather than replacing traditional methods, these digital resources should be thoughtfully integrated into a broader educational model that values both innovation and embodiment. A “hybrid” pedagogical mode may be implemented to bridge dissection and hybridity, by adopting a concise “see–touch–simulate–reflect” cycle. Learners begin at the donor table to develop haptic and spatial understanding of tissue planes and anatomical variation. They then add the hybrid layer, analyzing the tissue interface with 3D‐printed components, meshes, or implants, and make explicit how these alter landmarks and mechanics. Next, students work with probabilistic atlases or patient‐like digital twins to plan or test decisions, then map those choices back to the physical constraints observed at the table. A brief and structured debrief closes the loop and may include an engineer, physiotherapist, or ethicist to surface practical and ethical considerations.

## EMBODIMENT AND HYBRIDITY: FROM THE DONOR TABLE TO THE TECHNOLOGICAL BODY

Anatomical education has always extended beyond the acquisition of knowledge: it is also a space of confrontation with mortality, vulnerability, and the physical reality of the human condition. Dissection with donor bodies has historically been a formative experience for future physicians, fostering not only anatomical skill but also reflection, emotional maturity, and ethical awareness. Recent studies reinforce this perspective. An analysis of reflective essays by medical students found that dissection catalyzed deep personal growth, empathy, and resilience.^36^ Similarly, another study showed that even prior to interacting with a deceased donor, students expressed gratitude toward donors and recognized the ethical significance of their anatomical studies.^37^ Despite advances in technology, virtual simulations lack these formative dimensions. Some authors introduced the concept of “body pedagogics,” critiquing the disembodiment of modern medical education. They argue that while cognitive knowledge is essential, embodied learning, through physical engagement, sensory input, and emotional resonance, grounds students in the reality of human life and death.^38^ This perspective aligns with foundational philosophies of embodiment,^39^, ^40^ which situate perception and learning in lived, tactile experience, and empirical comparisons support this claim. Recently, Krebs and Hildebrandt have framed anatomy as a form of “embodied resistance” to digital abstraction, arguing that hands‐on engagement with human remains, that is grounded in the phenomenology of the lived body, counterbalances AI‐ and data‐driven models by nurturing tactile understanding, empathy, and ethical reflection.^41^ Accordingly, a recent survey of over 450 students found strong support for donor‐based dissection, especially among medical students, who valued its emotional and educational impact.^42^ Virtual anatomy tools, while optimized for interactivity and visualization, were seen as supplemental. These concerns were echoed by other work, warning that while AI and virtual dissection tables offer valuable tools, they may never fully replicate the tactile and variable experience of donor‐based learning.^42^ However, emerging studies show that well‐designed VR experiences, especially when paired with reflective debriefs, can also foster empathy and donor gratitude. For example Alieldin et al.,^43^ demonstrated measurable increases in learner empathy following immersive VR interventions, while Arthur et al.^44^ found that simulated conversations about donors deepened students' professional identity formation and appreciation of donor generosity. Therefore, the danger is not technology itself, but its uncritical adoption. Though based on real scans, digital tools tend to present as cleaned, idealized, and decay‐free versions of the body. This tension has been called the “simulation paradox” the idea that digital simulations, while controllable and replicable, eliminate the variability and uniqueness that make real human bodies educationally and ethically irreplaceable.^45^ It is also important to note that while some concerns have been raised about potential reductions in bedside empathy with overreliance on immersive tech^46^ robust longitudinal data are still limited, highlighting a current evidence gap that merits cautious interpretation. The recent development of the Cadaver Anatomy Soft Skills Scale (CASSS) found that dissection consistently fostered greater confidence, professionalism, and comfort with mortality than digital alternatives.^47^ In sum, embodied learning remains essential. Without tactile experience and the moral weight of physical presence, anatomical education risks becoming a sterile visual exercise. Hildebrandt and colleagues have proposed a new ethos for anatomical education, one that re‐centers the donor body as more than a teaching object, acknowledging its role in cultivating empathy, professional identity, and ethical awareness.^48^ Other authors have emphasized that anatomy provides much more than basic knowledge of structure and function; it serves as one of the earliest and most formative stages in healthcare training, fostering scientific understanding but also the development of key human competencies such as communication, ethical reasoning, and professional identity.^49^ These findings are further supported by a multi‐country qualitative study exploring anatomy educators' perspectives, which highlighted that body donor–based education uniquely supports the acquisition of non‐traditional, discipline‐independent skills (NTDIS), such as teamwork, cultural sensitivity, and ethical awareness, skills that are difficult to replicate through digital‐only modalities.^50^ Blended curricula, which preserve anatomical donors' learning while embracing digital tools, offer a path forward, one that prepares not only skilled technicians but reflective and compassionate clinicians. As we design the classrooms of the future, we must ask: What kind of anatomist, and what kind of professional, are we shaping through these new modalities?

**FIGURE 2:**
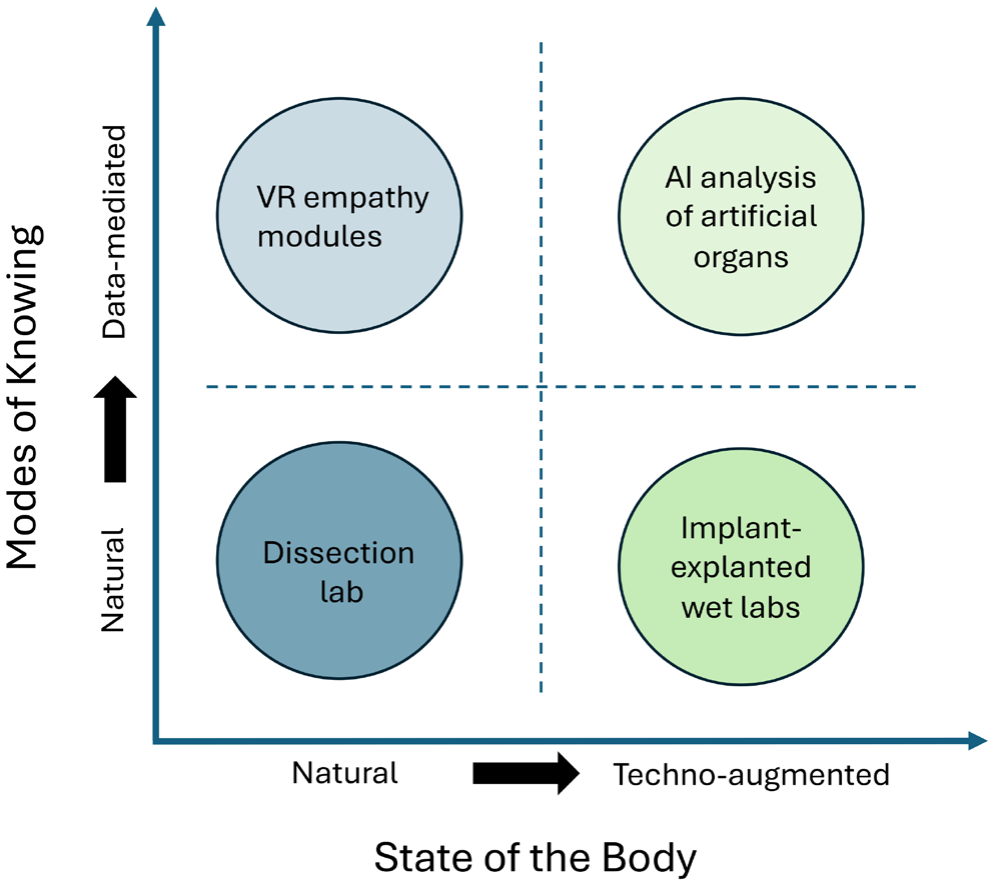
Conceptual framework linking embodiment and hybridity in anatomy education. The vertical axis charts the mode of knowing (from embodied to data‐mediated), while the horizontal axis charts the state of the body (from natural to techno‐augmented). Teaching activities are mapped onto this grid to illustrate their complementarity: Donor‐based dissection (natural), VR empathy modules (data‐mediated–natural), implant–explanted wet labs (natural–techno‐augmented), and AI analysis of artificial organs (data‐mediated–techno‐augmented). This model visualizes the continuum from tactile engagement with the natural body to technologically mediated interactions with hybrid bodies, underscoring the need for curricula that prepare learners to navigate both domains. Image created using PowerPoint software.

Embodiment is not an alternative to hybridity but its prerequisite. The sensory–cognitive coupling developed at the donor table (recognizing tissue planes, variability and resistance) transfers directly to device–tissue interfaces (e.g., mesh fixation, vascular grafts, and BCI leads). Materials literacy, feeling the differences among fascia, peritoneum, suture, and implant, prepares learners to anticipate altered biomechanics and complication patterns in augmented patients. Finally, the ethical and affective stance cultivated through donor work grounds consent, communication, and shared decision‐making with technologically modified patients. As we move toward increasingly hybrid bodies, the skills and sensibilities forged in embodied anatomical education become the foundation for navigating augmentation. This continuum from natural, embodied anatomy to technologically mediated, hybrid anatomy is conceptualized in Figure 2, where the *mode of knowing* (natural—data‐mediated) intersects with the *state of the body* (natural—techno‐augmented). Mapping teaching activities onto this space, such as donor dissection, VR empathy modules, and implant–explanted wet labs, illustrates their complementarity and clarifies how they collectively prepare learners for diverse anatomical realities.

The anatomical body we study, teach, and model may no longer represent a shared biological standard. Emerging biotechnologies are steadily transforming the human organism into a modifiable, customizable entity. Neural implants, bioengineered organs, brain–computer interfaces (BCIs), embedded sensors, and heritable genetic modifications are no longer speculative scenarios: they are entering clinical and experimental practice.^51^, ^52^, ^53^ As emerging augmentations transform the body, from restorative devices (e.g., neural implants and prosthetics)^54^ to enhancement‐oriented technologies (e.g., BCIs, robotic extensions, genome editing for treating rare, and/or hereditary diseases),^55^, ^56^ anatomy educators face new challenges. What bodies are we preparing our students to encounter, understand, or treat? Will our reference models continue to reflect real‐world patients or become increasingly obsolete in the face of technological diversity? As the body becomes more hybrid, we risk losing the physical, vulnerable, and real‐life experience that anatomy has always helped students to understand. These interventions range from sensory‐restorative to performance‐enhancing, each raising specific questions about what constitutes “normal” anatomy and function. The educational risk is not hybridization per se, but the dissociation from lived biological experience if such changes are taught solely through technological narratives. Without a grounding in the anatomical realities of vulnerability, decay, and individuality, students may lose the ability to critically engage with the ethical and clinical complexity of hybrid bodies. This concern echoes earlier issues raised with synthetic simulations: a visually idealized hybrid body, taught without reference to its organic basis, can flatten both anatomical and moral understanding. If we no longer teach anatomy as an engagement with the fallible, embodied human form, what becomes of our understanding of healing, suffering, and presence? From an ethical standpoint, enhancements through BCI, genome editing, or synthetic grafts challenge core definitions of identity and agency.^57^ The prospect of artificial chimerism and “biocyborgs” forces us to rethink not only anatomy curricula but also our normative concepts of the body.^54^

For anatomists, this represents both a challenge and an opportunity. Rather than becoming obsolete, anatomy may evolve into a learning space where future clinicians and health professionals learn to navigate and make sense of the hybrid body. In the broader context of surgical education, a growing body of evidence underscores the irreplaceable role of donor‐based training. In high‐precision specialties like robotic cardiac surgery, where procedures increasingly involve bioengineered implants that demand both technical finesse and anatomical familiarity, this hands‐on environment enables trainees not only to refine motor skills but also to adapt to the tactile realities of advanced materials and human variability, offering an authenticity not achievable through virtual or synthetic simulations. For instance, recent studies confirm that the use of donated human bodies remains irreplaceable in robotic cardiac surgery education, with evidence showing that it enables faster expert‐level proficiency than VR or other simulation methods, facilitates rapid mastery of complex skills in novice surgeons, and offers unmatched anatomical realism essential for advanced procedural training compared to synthetic or virtual models.^58^, ^59^ Integrating artificial implants, bioengineered tissues, and device‐tissue interactions into anatomical teaching is no longer optional but essential.^60^ This shift calls for a dual perspective: preserving rigorous knowledge of the natural body while extending the curriculum to encompass technological embodiment and its implications. Ultimately, as technologies redefine what the body can do and be, anatomy may be the only discipline still asking: What does it mean to be human when the human body is no longer entirely natural? This integrated view of embodiment and hybridity shapes the future of anatomical education; training that moves along this continuum, anchored in the tactile and ethical grounding of the donor table and attentive to the challenges of the hybrid body, positions anatomy as a critical human science, preparing students to face the ethical and professional questions of a rapidly transforming human condition.

## HUMAN ANATOMY AS A CRITICAL EDUCATION

In this rapidly transforming era, anatomy risks being seen as a static or outdated discipline, a preclinical step to be completed before “real” medical training begins. This perception is not only inaccurate; it is misleading and potentially harmful. As biomedical technologies begin to reshape the human body, anatomy becomes more relevant than ever. Teaching hybrid anatomy not only sharpens students' critical awareness of how bodies are increasingly entangled with devices and data, but also aligns with accreditation goals that emphasize professionalism, systems‐based practice, and interprofessional competence.^61^ Embedding such content prepares future clinicians to care for patients whose anatomies are technologically mediated through implants, prostheses, or algorithmically guided interventions,^62^ and invites collaboration with fields like bioengineering, physiotherapy, and occupational therapy that have long grappled with these hybrid realities. Grounding this approach in perspectives from bioethics and critical disability studies ensures that anatomy does not stand alone as the sole guardian of human meaning but participates in a broader responsibility to interrogate what it means to inhabit and care for altered bodies.^63^

Moreover, we believe that anatomy should be reclaimed as a critical human science: a place where students are encouraged not only to study form, but to reflect on what it means to live in a body during a time of deep transformation. The anatomy lab, whether physical or digital, can serve as a space of resistance to disembodiment. Here, the body will not be only measured and deconstructed but encountered and interpreted. In this context, anatomists are no longer just technical instructors. They become educators of critical awareness, helping students to understand where the biological and the artificial increasingly converge. Fulfilling this role requires expanding our pedagogical focus. Beyond structural knowledge, anatomy education should foster visual literacy, the ability to read the biological form with contextual, functional, and interpretive insight, not just pattern recognition.^64^ It should cultivate structural reasoning, helping students understand how the organization supports physiological function and how dysfunction or technological intervention alters this balance. Finally, it must encourage ethical reflection, engaging with the social, cultural, and spiritual dimensions of the human body, including its enhancement, modification, or hybridization.^65^ Understanding human anatomy remains essential, even when the body is no longer entirely natural. The anatomist's role is therefore both traditional and forward‐looking: preserving deep biological knowledge while preparing students to engage with the ethical and structural complexities of the hybrid human. At its core, anatomy offers a powerful lens through which to explore what it means to be human, particularly now. As our students prepare to work with bodies that may be edited, enhanced, or digitally rendered, we must help them ask not only how, but also why. What is gained, and what is lost, when the body changes? In this light, anatomy is more than a foundation for medicine: it is an anchor for human understanding, guiding education through a moment of unprecedented change. Beyond curricular design, these principles can be operationalized through targeted assessment strategies. Rubric items can explicitly evaluate students' ability to integrate anatomical knowledge with the functional and ethical dimensions of hybrid bodies. For example, explaining how a cochlear implant alters sensory affordances and identifying its anatomical seat; comparing the biomechanical implications of a synthetic vascular graft versus a native vessel; or anticipating changes in load distribution when a prosthetic joint is integrated into a limb and linking them to relevant anatomy. Such items help students move beyond memorization, applying anatomical reasoning to clinical contexts shaped by technology.

## CONCLUSION: TEACHING AT THE EDGE

This viewpoint does not aim to introduce entirely new concerns, but to offer an interpretive synthesis that connects long‐standing debates in anatomy education with the accelerating challenges of the hybrid human condition. It is a call for critical adoption of technological innovation, to embrace new tools without surrendering the core values of anatomical education. The future will undoubtedly bring more immersive technologies, more intelligent simulations, and more efficient delivery systems.^66^ But as anatomists, we must ensure that these do not come at the cost of presence, empathy, or human meaning. The body we teach is changing, not only in how it is presented in the classroom, but in what it is becoming in the real world. Implanted, edited, networked, and enhanced, the human form is shifting from biological fact to technological possibility.^67^ As Harry Parker powerfully suggests in his book *Hybrid Humans* (2022), “all humans are becoming hybrids,” whether through prosthetics, biohacking, or everyday technologies like contact lenses. His personal account highlights the urgency of rethinking anatomical education in an era where the body is no longer solely biological. We must prepare future professionals to not only locate structures, but to understand the ethical implications of acting upon them. In conclusion, to teach anatomy today and in the future is to guide students across a rapidly shifting world, where biology and technology increasingly converge. It is to hold the line of core knowledge, while opening the door to a deeper, more conscious understanding of what it means to be human.

## AUTHOR CONTRIBUTIONS

**Katia Cortese:** Conceptualization; writing – original draft; writing – review and editing. **Paola Falletta:** Conceptualization; writing – original draft; writing – review and editing.

## FUNDING INFORMATION

This work received no specific grant from any funding agency in the public, commercial, or not‐for‐profit sectors.

## CONFLICT OF INTEREST STATEMENT

The authors declare no conflicts of interest related to the content of this manuscript.

## Data Availability

Not applicable.
